# On a new *Dictyna* species (Araneae, Dictynidae) from the northern Palaearctic confused with the East Siberian *D. schmidti* Kulczyński, 1926

**DOI:** 10.3897/zookeys.138.1849

**Published:** 2011-10-19

**Authors:** Yuri M. Marusik, Niclas R. Fritzén

**Affiliations:** 1 Institute for Biological Problems of the North, Portovaya Str. 18, Magadan 685000, Russia; 2 Zoological Museum, University of Turku, FI-20014 Turku, Finland; 3 Klemetsögatan 7B7, FI-65100 Vasa, Finland

**Keywords:** spiders, Siberia, Palaearctic, Russia, Finland, epigyne, receptaculum

## Abstract

A new species, *Dictyna palmgreni* **sp. n.**, is described from Finland and Russia on the basis of both sexes. Most of the earlier records of *Dictyna schmidti* Kulczyński, 1926 from the northern Palaearctic refer to this new species. *Dictyna shilenkovi* Danilov, 2000, **syn. n**. from Cisbaikalia is synonymised with *Dictyna schmidti*. The general appearances and copulatory organs of *Dictyna palmgreni* **sp. n.**, *Dictyna schmidti* and *Dictyna major* Menge, 1869 are illustrated. The distribution of *Dictyna palmgreni* **sp. n.** and*Dictyna schmidti* is clarified. An unknown sac-like structure of the spermathecae of Dictyninae is briefly discussed.

## Introduction

Dictynidae is a globally distributed medium-sized family with 566 chiefly cribellate species belonging to 50 genera (Platnick 2011). The largest dictynid genus is *Dictyna* Sundevall, 1833. It encompasses 123 species distributed mainly in the Holarctic Region (Platnick 2011). Although *Dictyna* is a fairly large genus and its representatives are rather common, this genus has never been revised on a wide scale. The only detailed revision made for the Nearctic fauna is that by Chamberlin and Gertsch (1958).

The family, and the genus *Dictyna* particularly, is relatively well studied in northern Europe and Asia. Nevertheless, several species occurring in Siberia and northern Europe remain inadequately studied and are known from the original descriptions or from one sex only. The Siberian Dictynidae have been treated by (Kulczyński (1908, 1916, 1926), Marusik (1988), (Danilov (1994, 2000) and Marusik and Koponen (1998).

The species *Dictyna schmidti* was described from Kamchatka by Kulczyński (1926) on the basis of a single male. Later this species was redescribed on the basis of Finnish specimens (Lehtinen 1967). Reasoning from Lehtinen’s illustrations, this species was reported from other localities in Finland and adjacent Russia (Palmgren 1977) and Sweden (Pettersson 1996, Almquist 2006). *Dictyna schmidti* was also reported from several localities in the Urals (see references in Esyunin and Efimik 1996) and Siberia (see Mikhailov 1997, Danilov 2000). While studying spiders of Siberia and Finland we have found specimens that match Lehtinen’s (1967) and Palmgren’s (1977) illustrations of *Dictyna schmidti*. Yet, we have found a few specimens from eastern Siberia that clearly differ from *Dictyna schmidti* sensu Lehtinen (1967) but well match Kulczyński’s description. A comparison of these specimens led us to the conclusion that the widespread species (from Fennoscandia to eastern Siberia) known earlier as *Dictyna schmidti* in fact belongs to a new species, the description of which is the main goal of this paper.

## Material and methods

Specimens were photographed using either a JEOL JSM-5200 scanning electron microscope or an Olympus E-520 camera attached to an Olympus SZX16 stereomicroscope at the Zoological Museum, University of Turku. Drawings were made either by using a grid method with a MBS-9 stereomicroscope or a Leitz stereomicroscope with a camera lucida. Macerated epigynes were temporarily coloured with Chlorazol Black to make some parts more visible. Photographs were taken with specimens in dishes with alcohol and paraffin on the bottom. Holes of different sizes were made in the paraffin to keep the specimens in the appropriate position. The epigynes were macerated either with KOH solution or lactic acid. All measurements are in mm.

**Acronyms for depositories:** Zoological Museum, University of Turku, Finland (ZMT); Zoological Museum, University of Helsinki, Finland (ZMH); Zoological Museum of the Moscow State University, Russia (ZMMU); Swedish Museum of Natural History, Stockholm, Sweden (NHRS); Perm State University, Russia (PSU); Institute for Biological Problems of the North, Magadan, Russia (IBPN); Institute for Systematic and Ecology of Animals, Novosibirsk, Russia (ISEA); private collection of the second author, Vasa, Finland (NRF).

## Species survey

### Dictyna palmgreni sp. n.

urn:lsid:zoobank.org:act:EE0F36A3-845C-4667-A447-84C10B75AF2F

http://species-id.net/wiki/Dictyna_palmgreni

**Figures 1–5.**
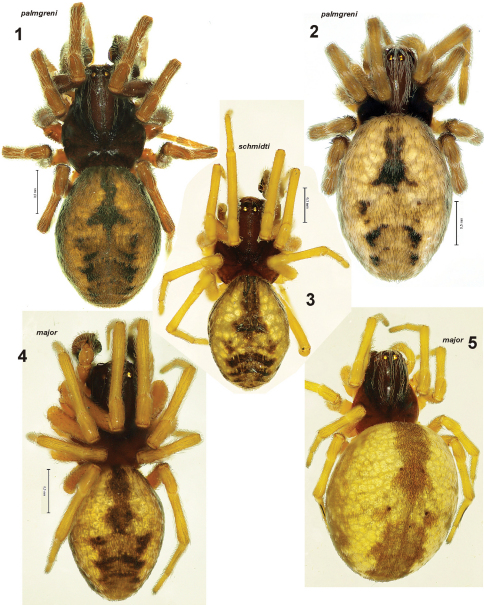
Habitus of *Dictyna palmgreni* sp. n. **1–2** from Pallastunturi, *Dictyna schmidti* **3** from Yakutia and *Dictyna major* **4–5** from Pyhtää. **1, 3–4** male; **2, 5** female.

Figs 1–2 6–9 12–13 18–19 22–23 28–30 32–33 40

Dictyna schmidti : Lehtinen 1967: 451, f. 292, 306; 452, f. 321 (♂♀).
Dictyna schmidti : Palmgren 1977: 21, f. 4.7-9 (♂♀).
Dictyna schmidti  (sensu Lehtinen): Danilov 2000: 42, f. 15-16 (♀).

#### Faunistic references

*Dictyna cf. major*: Marusik et al. 1992: 137.

*Dictyna* sp.: Marusik et al. 1993: 71.

*Dictyna schmidti* (sensu Palmgren): Esyunin & Efimik 1996: 136.

*Dictyna schmidti* (sensu Lehtinen): Pettersson 1996: 224.

*Dictyna schmidti* (sensu Lehtinen): Logunov et al. 1998: 131.

*Dictyna cf. schmidti*: Marusik et al. 2000: 21.

*Dictyna schmidti*: Almquist 2006: 315 (possibly misidentification).

#### Etymology.

 The specific name is a patronym in honour of the late Prof. Pontus Palmgren (1907–1993) who made a great contribution to studies of Finnish spiders.

#### Material examined.

 **FINLAND:** **Holotype** ♂ (ZMT), Muonio, Pallastunturi national park (np), SE slope of Laukukero, 68°02'53"N, 24°03'25"E, 31.05.2008, beaten from lower spruce branches at alpine tree line (N.R. Fritzén). **Paratypes:** 1♀ 4j (ZMT), same data as holotype; 2♂ 3♀ 9j (ZMT), Muonio, Pallastunturi np, 67°58'50"N, 24°04'23"E, 29.05.2007, spruce fen, at the border of a small open bog, beaten from lower spruce branches (N.R. Fritzén); 1♂ (ZMT), Muonio, Pallastunturi np. 67°58'47"N, 24°04'23"E, 29.05.2007, small semi open bog, sweeping (N.R. Fritzén); 2♂ 1♀ 4j (ZMT), Muonio, Pallastunturi np, SE slope of Laukukero, 68°02'52"N, 24°03'35"E, 27.05.2007, beaten from lower spruce branches near alpine tree line (N.R. Fritzén); 1♀ (ZMT/VR90), Kittilä, Alakylä, 67°21'N, 24°53'E, 17.06.1963 (P.T. Lehtinen) (referred to as allotype of *Dictyna schmidti* in Lehtinen (1967) and Palmgren (1977); 1♀ 1j (ZMH), Muonio, kirkonkylä, 67°56'N, 23°41'E, 14.07.1943, swampy forest (P. Palmgren); 1♂ (ZMH), Kittilä, 67°39'N, 24°54'E, ?1857 (Nylander & Gadd) (labelled as *Dictyna schmidti* ssp. *abieticola* ♂ holotype by P.T. Lehtinen); 1♂ (ZMH), Kalajoki, Pentti isl., 64°11'14"N, 23°41'53"E, 8.07.1999, pitfall trap in mesic heath forest with dense stand of *Picea abies*, (M. Sievänen). **RUSSIA**: ***Murmansk*** Area: 3♀ 5j. (ZMH) (Lt) Lotta river, 50 km E of Finnish frontier, 9.08.1967 (M. Meinander). ***North Urals***:2♀(NHRS), Vishorski Reserve, Ol’khovka River, forest, 13.7.1994 (O. Garkunova). ***Middle Urals*:** 6♀ (PSU), Basegi Mnt., forest, branches of *Picea*, 1.09.1990 (S.L. Esyunin). ***Yamal Peninsula*:** 3♀ (2 with missing epigynes) (PSU), *South Yamal*, Khadyta-Yakha River, mixed forest, 06.1982 (S.L. Esyunin). ***Krasnoyarsk*** Province: 1♂ (ISEA), West Sayany, south macroslope of Oiskiy Mt. range, 11 km S of Oiskoye Lake, Buiba River valley, 52°47'N, 93°18'E, 1200-1230 m, 20-21.06.1995 (A. Abramov). ***Yakutia*:** 1♂3♀ (ZMMU), Yakutia, Lena River, 10 km downstream off Zhigansk, mouth of Ynyr Khaya Spring, stony bank and meadows, 4-8.07.1989 (K.Yu. Eskov). ***Magadan*** Area:1♂ (ZMMU), Upper Kolyma flow, Sibit Tyellakh River basin, Olen’ River valley, environs of “Aborigen” Field Station, on ice field, 600 m, 7.06.1985 (Yu.M. Marusik).

#### Diagnosis.

 *Dictyna palmgreni* sp. n. resembles *Dictyna major* and *Dictyna schmidti*, from which it can be easily separated by the shape of the apical portion of the conductor (broadening and then abruptly tapering, not gradually tapering like in the other two species), the relatively short cymbium, the thick and spiralled epigynal ducts and also by the presence of a digitiform process (accessorial gland). In the male palp, the combination of short length and basal placement of the tibial apophysis also distinguishes it from the two other species.

#### Description.

Male. Total length 2.63-3.00. Carapace: 1.10-1.30 long, 0.88-0.95 wide, cephalic part 0.60 wide, clypeus 0.14, chelicerae 0.79. Abdomen 1.75 long, 1.20 wide. Cymbium 0.69-0.79 long, 0.40-0.43 wide, length/width ratio 1.70-1.80. Leg I segments: femur 1.17, patella+tibia 1.36, metatarsus 0.86, tarsus 0.57. Carapace brown, cephalic part raised, well separated from thoracic part by ‘furrow’, cephalic portion with 5 longitudinal ‘furrows’ with sparse whitish hairs, thoracic part with radial stripes. Abdomen light to dark brown with dark grey-brownish pattern (Figs 1, 7), somewhat variable and sometimes with cardiac mark posteriorly trifid. Palp as in Figs 18-19, 22-23, tibia short, apophysis carrying ctenidia short (about 2 lengths of ctenidia) and positioned near base of tibia; conductor in one plain, upper arm of conductor abruptly cut, lower arm with bent thin tip directed retrolaterad.

Female. Total length 2.90-3.10. Carapace: 1.05-1.18 long, 0.91-0.94 wide, brown with dark-grey radial stripes, and light brown median band (behind posterior eye row). Cephalic portion with 5 longitudinal ‘furrows’ densely covered with whitish hairs. Clypeus 0.13, chelicerae 0.60. Leg I segments: femur 1.07, patella+tibia 1.14, metatarsus 0.69, tarsus 0.50. Abdomen light brownish with brown pattern as in Figs 2, 8-9, usually with cardiac mark posteriorly distinctly trifid, venter with median dark band. Epigyne as in Figs 28-30, 32-33 with thin septum and rather long margins. Vulvae with spiralled insemination ducts terminated by spiralled ‘receptacula’. ‘Receptacula’ with digitiform cylindrical accessorial gland.

**Figures 6–11.**
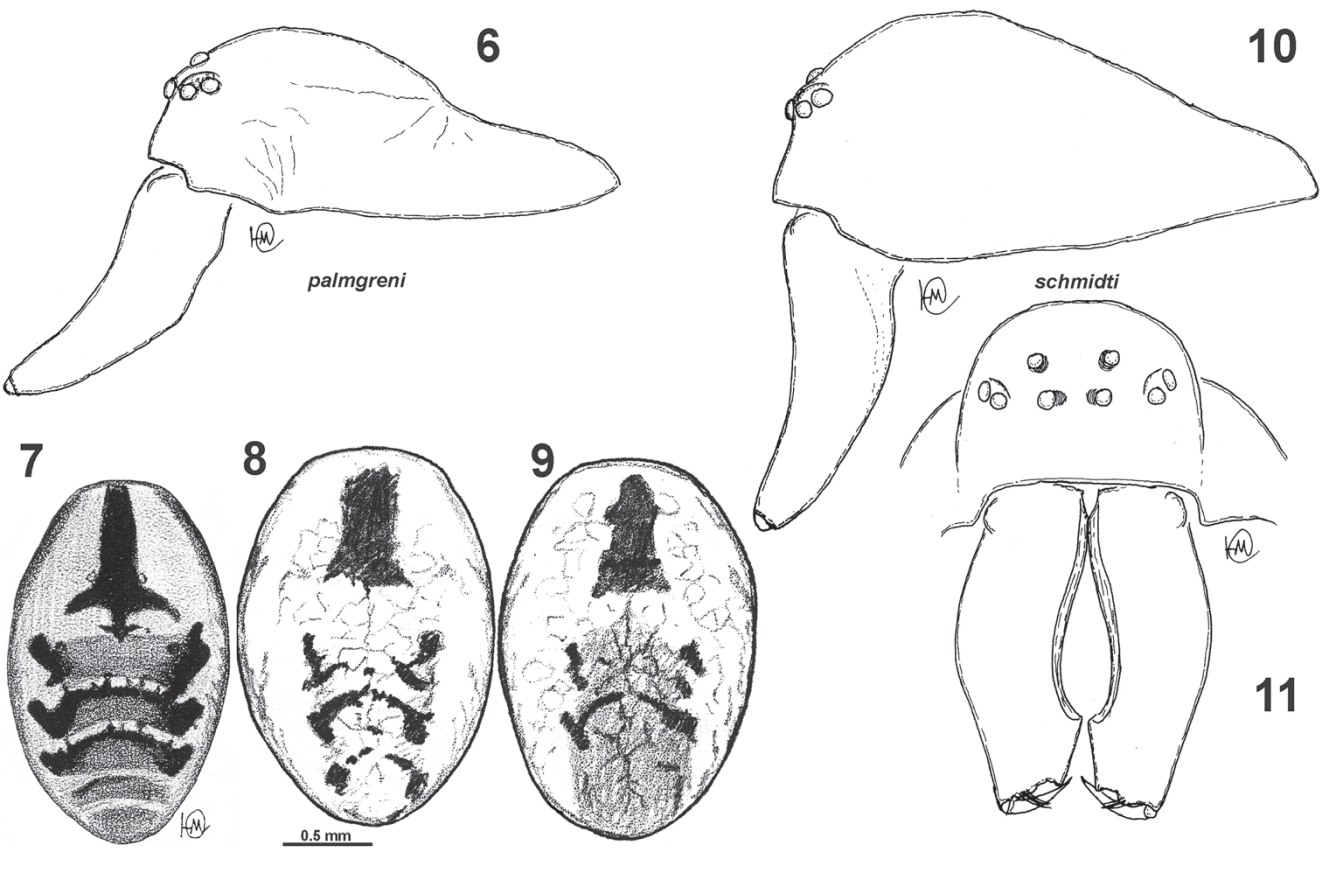
Prosoma and abdomen of *Dictyna palmgreni* sp. n. **6–9** and *Dictyna schmidti* **10–11**. **6, 10** – male carapace, lateral **7** male abdomen, dorsal **8–9** female abdomen, dorsal **11** prosoma, frontal **6 10–11** from the Upper Kolyma **7** from Krasnoyarsk Province **8–9** from Basegi (Ural).

**Figures 12–17.**
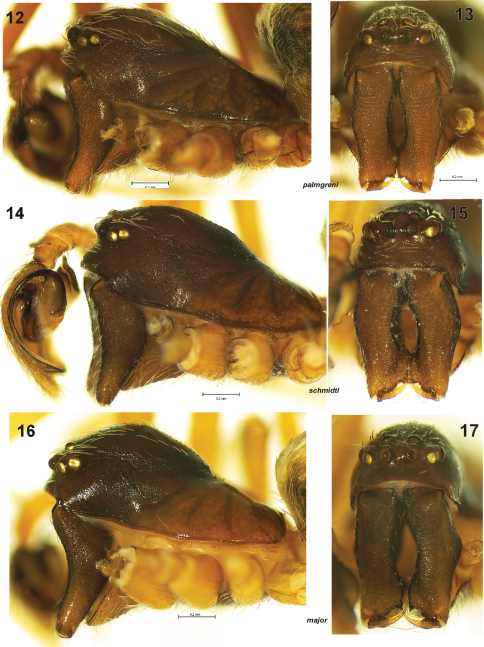
Male prosoma of *Dictyna palmgreni* sp. n. **12–13** from Pallastunturi, *Dictyna schmidti* **14–15** from Yakutia and *Dictyna major* **16–17** from Pyhtää **12, 14, 16** lateral **13, 15, 17** frontal.

**Figures 18–21.**
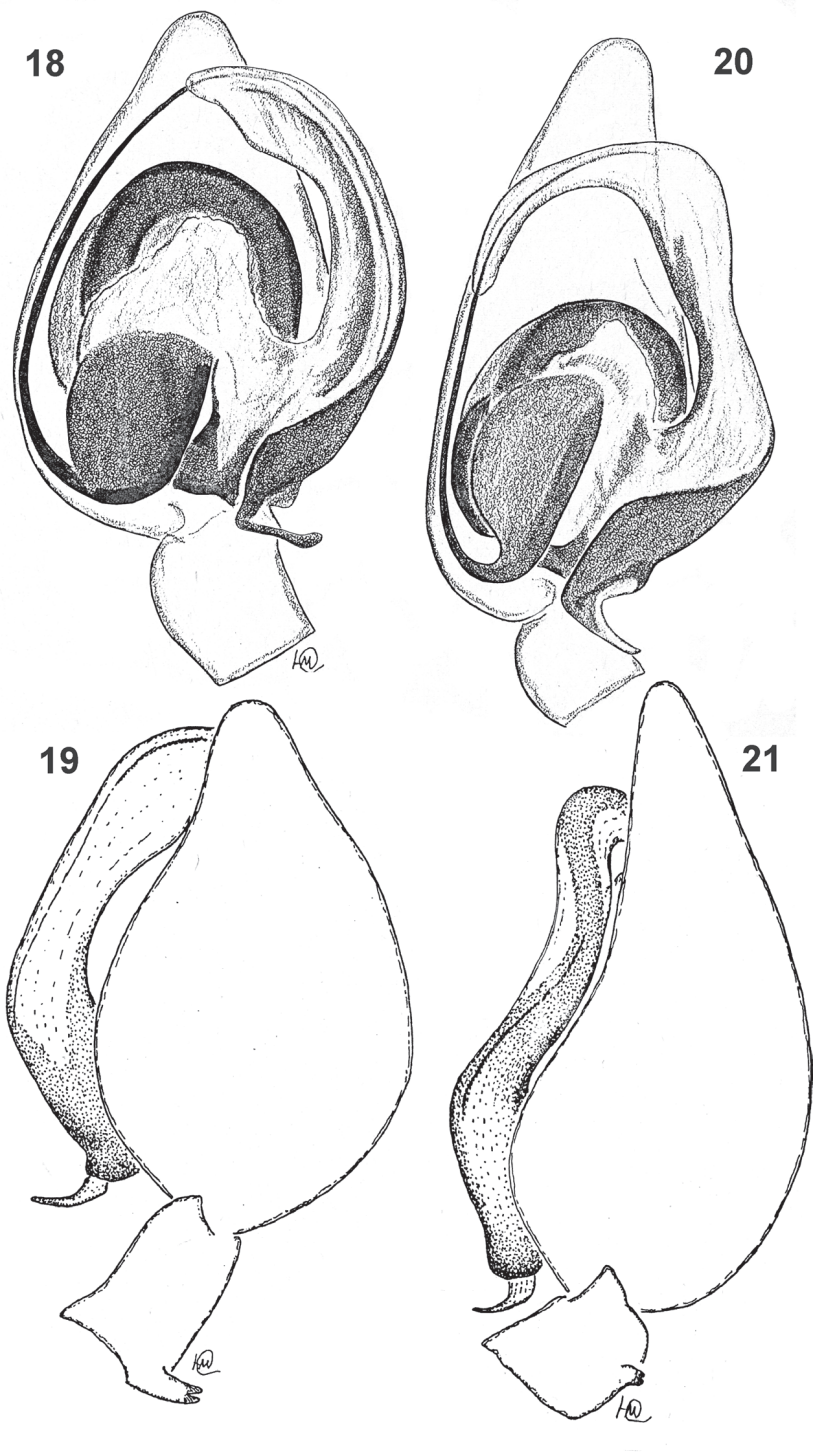
Male palp of *Dictyna palmgreni* sp. n. **18–19** and *Dictyna schmidti* **20–21** from the Upper Kolyma **18, 20** ventral **19, 21** retrolateral.

**Figures 22–27.**
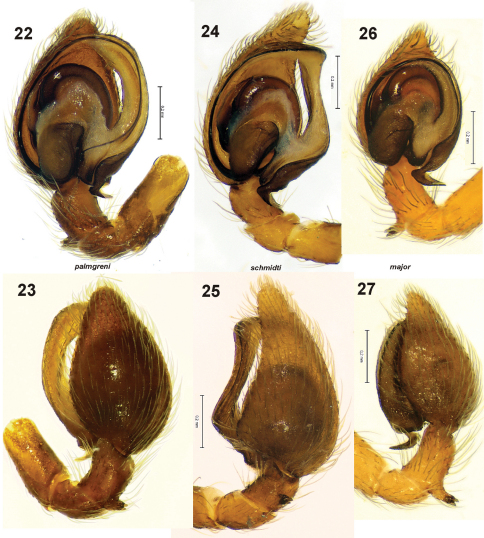
Male palp of *Dictyna palmgreni* sp. n. **22–23** from Pallastunturi, *Dictyna schmidti* **24–25** from Yakutia and *Dictyna major* **26–27** from Pyhtää **22, 24, 26** ventral **23, 25, 27** retrolateral.

**Figures 28–31.**
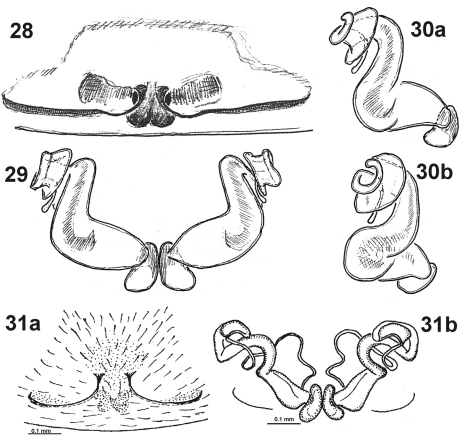
Epigyne of *Dictyna palmgreni* sp. n. **28–30** from Basegi (Ural) and *Dictyna schmidti* **31.** **28, 31a** epigyne, ventral **29, 31b** sclerotised part of receptacula **30a,b** left receptaculum, different aspects. **31** after Danilov (2000), sub. *Dictyna shilenkovi*.

**Figures 32–39.**
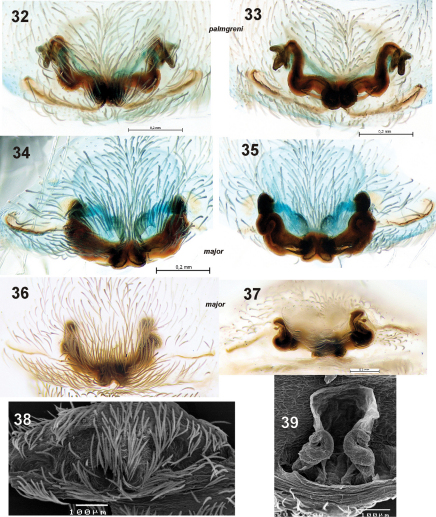
Epigyne of *Dictyna palmgreni* sp. n. **32–33** from Pallastunturi and *Dictyna major* **34–39** from Pyhtää **32, 34, 36, 38** macerated epigyne, ventral **33, 35, 39** macerated epigyne, dorsal **37** macerated epigyne showing sac-like structure, frontal. Sac-like structure on Fig. **39** collapsed and sclerotised parts of epigyne became closer.

**Figure 40.**
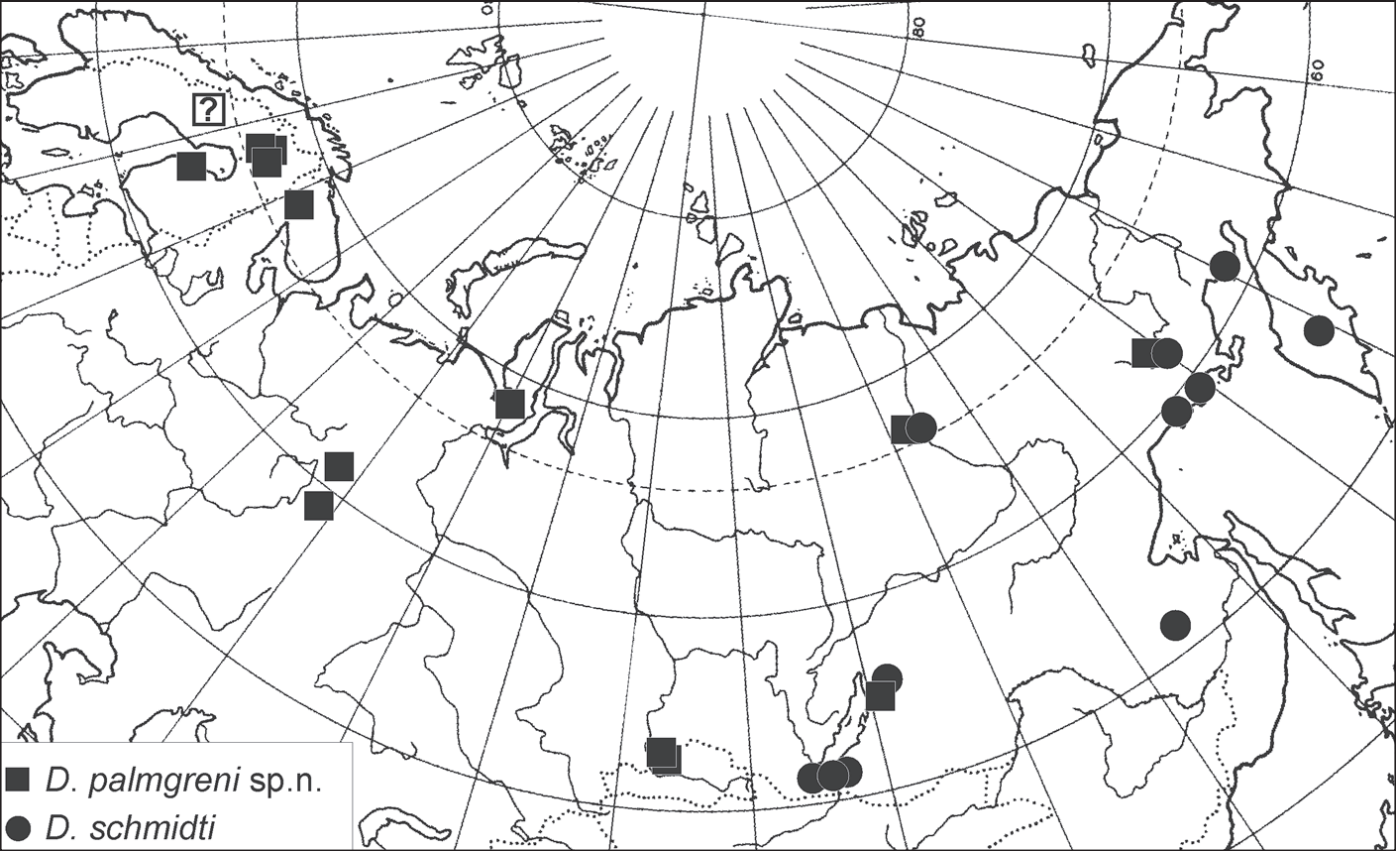
Distribution map of *Dictyna palmgreni* sp. n. (square) and *Dictyna schmidti* (dot).

#### Distribution.

 The new species is known across almost the entire northern Palaearctic: from Fennoscandia to Magadan, north to 68° in Finland, and southward to about 53° in Krasnoyarsk Province of Russia. To date, there have apparently been no documented adult specimens from Sweden (L. Jonsson & R. Pettersson pers. comm.), which are needed for the confirmation of its occurrence there.

#### Natural history.

 Adult females occur from late May throughout the summer, males from late May to at least the beginning of July. Finnish specimens have mainly been collected from stands dominated by Norway spruce (*Picea abies*), and often on moist ground (swampy forest or mires). At least to some extent the species is arboreal, but some specimens have been caught using pitfall-traps and some apparently live in open habitats.

### Dictyna schmidti

Kulczyński, 1926

http://species-id.net/wiki/Dictyna_schmidti

Figs 3 10–11 14–15 20–21 24–25 31 40

Dictyna schmidti  Kulczyński, 1926: 37, pl. 2, f. 1-3 (♂; the ♂ holotype not examined).
Dictyna shilenkovi  Danilov, 2000: 42, f. 17-20 (♂♀), syn. n. (the ♂ holotype not examined).

#### Faunistic references

*Dictyna schmidti*: Marusik et al. 1992: 137.

*Dictyna schmidti*: Marusik et al. 1993: 71.

*Dictyna schmidti*: Marusik 1988: 1482; 2005a: 266; 2005b: 190.

*Dictyna shilenkovi*: Trilikauskas 2008: 38.

#### Remarks.

The ♂ holotype from Klutschevskoje, Kamchatka retained in the Institute of Zoology PAN (Warsaw, Poland) has not been found. The ♂ holotype of *Dictyna shilenkovi* and two ♀ paratypes indicated as being deposited in the Zoological Museum of the Moscow State University (see Danilov 2000) have not been found there.

#### Material examined.

 **RUSSIA**: ***Yakutia*:** 1♂ (IBPN), c. 10 km downstream of Zhigansk, mouth of Ynyr Khaya Spring, 4-8.07.1989 (K.Yu. Eskov). ***Magadan*** Area: 1♂, 50 km N of Magadan, Khasyn River valley near Splavnaya Village, 28.05.1988 (Yu.M. Marusik & S.A. Ryabukhin); 1♂ (IBPN), upper Kolyma River flow, Sibit Tyellakh River basin, Olen‘ River valley, environs of “Aborigen” Field Station, around ice field, sweeping grass near alder stand, h 650m, 27.07.1987 (Yu.M. Marusik); 1♂ (IBPN), 180 km W of Magadan, Cholomdzha River middle flow, 1988 (N.Y. Dokuchaev); 1♂, Taigonos Peninsula, Paren‘ River middle flow, 07.1985 (A. Meshcheryakov).

#### Description.

Male. For details see Kulczyński (1926) and Danilov (2000: sub. *Dictyna shilenkovi*). Carapace 1.35 long, 1.07 wide, cephalic part 0.52 wide. Chelicerae 0.75 long. Leg I segments: femur 1.15, patella & tibia 1.43, metatarsus 0.91, tarsus 0.58. Abdomen 1.60 long, 1.10 wide. Palp as in Figs 20-21, 24-25; process carrying ctenidia located in mid part of tibia, very small; conductor long, three-dimensional (not in one plain), its apical arm gradually tapering and terminating on prolateral side, lower arm small and directed retrolaterad-backward.

Female. Described by Danilov (2000: sub. *Dictyna shilenkovi* ). Paratypes have not been available for this study. Epigyne (Figs 31a-b) with thin sclerotized parts of receptacula.

#### Distribution.

 This species is known from East Siberia only (Fig. 40): from Transbaikalia, northward to Zhigansk, southward to Ulan-Ude (Buryatia) and Bureinski Reserve (Khabarovsk Province) and eastward to Kamchatka.

#### Natural history.

 One specimen was collected by sweeping grasses on a north exposed slope in the Upper Kolyma. One male near Magadan was found under stones. The type specimens of *Dictyna shilenkovi* were mainly collected from mixed forests (Danilov 2000).

### Dictyna major

Menge, 1869

http://species-id.net/wiki/Dictyna_major

Figs 4–5 16–17 26–27 34–39

Dictyna major : Wiehle 1953: 100, f. 218-221 (♂♀).
Dictyna major : Chamberlin & Gertsch 1958: 82, pl. 24, f. 2-4 (♂♀).
Dictyna major : Roberts 1985: 50, f. 14c (♂♀).
Dictyna major : Roberts 1995: 84, f. (♂♀).
Dictyna major : Roberts 1998: 86, f. (♂♀).
Dictyna major : Paquin and Dupérré 2003: 68, f. 563-565 (♂♀).
Dictyna major : Almquist 2006: 314, f. 274a-g (♂♀).
Dictyna schmidti : Almquist 2006: 316, f. 276a-d (♂♀) (seems a misidentification). For a complete list of references see Platnick (2011).

#### Material examined.

 **FINLAND:** 1♂ 8♀ 1j (NRF), Pyhtää, Kaunissaari 60°21'42"N, 26°46'50"E, dune shore with sparse *Leymus arenarius*, 9.06.2009 (N.R. Fritzén); 1♂ (NRF), Lohtaja, Vattajanniemi 64°00'34"N, 23°23'26"E, in vegetation on dune shore, 7.06.2010 (N.R. Fritzén); 1♂ Kalajoki, Letto 64°17'02"N, 23°52'32"E, dune shore with sparse vegetation, 8.06.2010 (N.R.Fritzén); 1♂ 1♀ (ZMT) Utsjoki, Lohva, 12.07.1962 (P.T. Lehtinen). **CANADA**: **Yukon** Territory: 3♂ 4♀ (IBPN) Kluane Lake, environs of research station, south bank of the lake, 5-11.07.1993 (Yu.M.Marusik); 1♀ (IBPN) environs of Carmacks, 135º55'W, 62º04'N, steppe slope and surroundings, 18.07.1993 (Yu.M.Marusik).

Numerous specimens from Tuva (Marusik et al. 2000), North-East Siberia (Marusik et al. 1992), Yakutia (Marusik et al. 1993), Greenland (Marusik et al. 2006) have also been examined.

#### Comments.

 It has not been possible to trace the Finnish specimens used for making the figures of *Dictyna schmidti* in Almquist (2006). The illustrations are probably based on misidentified specimens and seem to refer to *Dictyna major*.

#### Description.

 Thoroughly described by Wiehle (1953), Chamberlin & Gertsch (1958) and Almquist (2006). Here we provide only comparative figures of the copulatory organs in order to demonstrate differences between it and the similar-looking *Dictyna palmgreni* sp. n. and *Dictyna schmidti*.

#### Distribution.

 The species has a circum-Holarctic range and is known across the Palaearctic and Nearctic Regions.

#### Natural history.

 This species has different habitat preferences in Siberia and in Finland. In Magadan Area, it is the most common dictynid species, occurring in various habitats within the forest belt and is most numerous on *Ledum* shrubs. In Finland, *Dictyna major* is rare, has a scattered distribution and occurs exclusively on dune shores.

## Relationships

Studying the relationships between *Dictyna* and the related *Emblyna* Chamberlin, 1948 faces certain difficulties. Both genera are species diverse, especially in the Nearctic, and their proper revisions in the Holarctic are lacking. Besides, data on the internal structure of the epigyne of the majority of Nearctic species is also lacking. Although males of the three species *Dictyna major*, *Dictyna palmgreni* sp. n. and *Dictyna schmidti* have similar palps, it is not clear whether they are related or not. The epigynes of these species are rather different. The copulatory openings of *Dictyna major* and *Dictyna palmgreni* sp. n. are similar, but those of *Dictyna palmgreni* sp. n. have a unique digitiform process of receptaculum which is absent in other *Dictyna* species known to us. The epigyne of *Dictyna schmidti* differs significantly from both *Dictyna major* and *Dictyna palmgreni* sp. n. The male palp of *Dictyna schmidti* and *Dictyna szaboi* Chyzer, 1891 1891 (cf. Gajdos and Pekár 1999: figs. 1-4) is also rather similar, both having a very long 3-dimensional conductor and a small tibial dorsal process. The vulva of *Dictyna szaboi* has never been illustrated.

## Notes on the structure of the internal part of epigyne in Dictyninae

While studying the epigynes of *Dictyna palmgreni* sp. n., *Dictyna major* and some other *Dictyna* and *Ajmonia* species we have found large transparent sac-like structures (cf. Figs 32-35, 37, 39; Figs. 5, 21 in Marusik and Koponen 1998; Fig. 2 in Marusik et al. 2006; Fig. 1i in Marusik and Esyunin 2010). Other authors have never reported on such structures. When we had prepared a specimen for making SEM photographs and transferred it from alcohol to a filter paper for drying up, the sac-like structure resembled a plastic bag, which immediately collapsed as soon as the filter paper was touched (cf. Fig. 39). Considering the very small size of the *Dictyna* receptacula, it seems that the sac-like structure serves as an additional unpaired receptaculum. We do not know any similar structures in other families belonging to the RTA-clade. Somewhat similar, unpaired transparent receptacula are known in Dysderidae, Oonopidae and the related haplogyne families (Figs 830-835 in Forster and Platnick 1985), but these are situated below the epigastric furrow and behind the unpaired “receptaculum”. In Dictyninae, the sac-like structure is situated between the integument and the paired receptacula.

## Supplementary Material

XML Treatment for Dictyna palmgreni

XML Treatment for Dictyna schmidti

XML Treatment for Dictyna major
